# The Alpaca *Melanocortin 1 Receptor*: Gene Mutations, Transcripts, and Relative Levels of Expression in Ventral Skin Biopsies

**DOI:** 10.1155/2015/265751

**Published:** 2015-01-05

**Authors:** Bathrachalam Chandramohan, Carlo Renieri, Vincenzo La Manna, Antonietta La Terza

**Affiliations:** ^1^KVASU Centre for Wildlife Studies, Kerala Veterinary and Animal Sciences University, Pookode, Wayanad, Kerala 673576, India; ^2^School of Biosciences and Veterinary Medicine, University of Camerino, Via Gentile III da Varano, 62032 Camerino, Macerata, Italy

## Abstract

The objectives of the present study were to characterize the *MC1R* gene, its transcripts and the single nucleotide polymorphisms (SNPs) associated with coat color in alpaca. Full length cDNA amplification revealed the presence of two transcripts, named as F1 and F2, differing only in the length of their 5′-terminal untranslated region (UTR) sequences and presenting a color specific expression. Whereas the F1 transcript was common to white and colored (black and brown) alpaca phenotypes, the shorter F2 transcript was specific to white alpaca. Further sequencing of the *MC1R* gene in white and colored alpaca identified a total of twelve SNPs; among those nine (four silent mutations (c.126C>A, c.354T>C, c.618G>A, and c.933G>A); five missense mutations (c.82A>G, c.92C>T, c.259A>G, c.376A>G, and c.901C>T)) were observed in coding region and three in the 3′UTR. A 4 bp deletion (c.224 227del) was also identified in the coding region. Molecular segregation analysis uncovered that the combinatory mutations in the *MC1R* locus could cause eumelanin and pheomelanin synthesis in alpaca. Overall, our data refine what is known about the *MC1R* gene and provides additional information on its role in alpaca pigmentation.

## 1. Introduction

Coat color in mammals depends on the synthesis and distribution of the relative amounts of eumelanin and pheomelanin, which are influenced by more than 350 genes [1]. The single exon gene* MC1R* has recently received much attention it encodes for the melanocortin 1 receptor (MC1R), which is a G-protein coupled receptor [2] specifically expressed by melanocytes. MC1R is a seven-transmembrane protein that plays a crucial role in melanogenesis stimulation upon binding to its physiological ligand agouti/*α*-MSH [3, 4]. In mammals and birds, increased MC1R activity enhances the production of eumelanin (dark, brown/black pigment), whereas decreased MC1R activity results in the production of pheomelanin (yellow/red pigment) [5, 6]. The* MC1R* gene was cloned at the beginning of the 1990s and has since been established as a major determinant of skin and hair pigmentation. Great efforts have been made to extensively genotype animals for useful information and associations with different coat color.* MC1R* has been extensively studied in mammals including mouse [5], cattle [7], horse [8], fox [9], sheep [10, 11], dog [12–14], rabbit [15], chicken [16], fish [17], and to some extent alpaca [18, 19].

Furthermore, there is a lack of information regarding* MC1R* molecular segregation, cDNA structure, and expression in different colors, which would reveal the mechanisms behind pigmentation. In this study, we report the cloning and characterization of* MC1R* full length transcripts and their relative levels of expression in white and colored (black and brown) Peruvian alpaca skin samples using RT-PCR analysis. These results will help to reveal how the* MC1R* gene is regulated in varying alpaca coat colors.

## 2. Materials and Methods

### 2.1. Collection and Storage of Skin Biopsies

Skin biopsies from white and colored (brown and black) alpacas were collected in March 2008 by disposable biopsy punch (8 mm diameter) in RNAlater (SIGMA, Germany). Alpacas were from the ILPA-Puno, Quimsachata Experimental Station, Instituto Nacional de Innovacion Agraria (INIA), Peru, which is located at 4300 m.a.s.l. The alpacas analyzed in the present study were part of a previous phenotypic segregation study on coat color inheritance [20]; these animals were also used for the molecular characterization of the* agouti* gene [21].

In respect to other countries, such as the USA and Australia, Peru accounts for about 90% of the worldwide alpaca population; thus this South-American country can be considered the largest reserve of alpaca biological resources in the world. All sampled white alpacas possessed only dark and not blue eyes. Peruvian breeders particularly consider the blue-eyed white phenotype as a defect and/or an undesirable trait and thus these animals are excluded from reproduction [22]. The biopsies were transferred to the School of Environmental Science, the University of Camerino, Camerino, Italy. Subsequently, the biopsies were removed from the RNAlater, blotted with sterile blotting paper, and stored at −196°C (liquid nitrogen) for further analysis. All experiments were approved and performed according to the guidelines of the Animal Ethics Committee of the University of Camerino.

### 2.2. Nucleic Acid Extraction and cDNA Synthesis

Total RNAs from stored skin biopsies were extracted using the RNAeasy fibrous tissue mini kit (Qiagen S.A., Courtaboeuf, France) and treated with RNase-free DNase to remove contaminant genomic DNA according to the manufacturer's instructions. Genomic DNA was also isolated using the DNAeasy tissue kit (Qiagen S.A., Courtaboeuf, France) according to the manufacturer's instruction. The quality and quantity of isolated RNA and DNA were measured using a GENESYS 10 UV spectrophotometer (Thermo, USA) and by calculating the ratio of optical density at A260/A280. RNA and DNA integrity were checked using 1.5% formamide-agarose gel electrophoresis and 0.8% agarose gel electrophoresis, respectively. RNA and DNA samples with good quantity and quality were stored at −80°C for further analysis. The first strand cDNA was synthesized using 2 *μ*g of total RNA with 10 pmol OdTm primer (Table 1), 0.5 mM dNTPs, 1 × RT buffer, 20 U RNase inhibitor, and 200 U PrimeScript Reverse Transcriptase (Takara Biotech, Japan) in a 20 *μ*L total reaction volume according to the manufacturer's instructions. The reaction mixture was incubated for 45 min at 50°C and then at 70°C for 15 min; the resulting cDNA was used in coding sequence and 3′UTR amplification. All PCR reactions were carried out using a Perkin-Elmer Thermal Cycler (Perkin-Elmer Corporation, Norwalk, CT, USA).

### 2.3. Primer Design and PCR Amplification of Full Length cDNAs

Orthologous sequences of the* MC1R* gene from mammals were retrieved from the NCBI GenBank (http://www.ncbi.nlm.nih.gov/) and aligned using EMBL ClustalW (http://www.ebi.ac.uk//Tools/clustalw/) to identify conserved regions for the design of primers for coding region amplification. PCR amplification of the complete coding sequence (CDS) was carried out with the forward (MCfw) and reverse (MCR5R1) primers (Table 1). Amplification of* MC1R* cDNA was performed at 95°C for 3 min, followed by 30 cycles of 95°C for 30 sec, 62°C for 30 sec, and 72°C for 1 min, with a final extension at 72°C for 7 min. Next, 5′ rapid amplification of cDNA end (RACE) was carried out as previously reported by [23] using the SA, ASA, and reverse MCR5R3 primers (Table 1). The 3′ RACE amplifications were completed using the NSTodt primer and a specific forward primer (MC1RFw) (Table 1). The PCR reaction included an initial denaturation step of 3 min at 95°C, followed by 35 cycles of denaturation at 95°C for 30 s, annealing at 62°C for 30 s, and extension at 72°C for 1 min 30 sec, with a final extension at 72°C for 7 min. All PCR amplifications were carried out in a final 50 *μ*L PCR reaction mixture containing 1 × Expand High Fidelity PCR System buffer (1.25 mM MgCl_2_), 0.3 mM dNTP, 0.3 *μ*mol of each primer, and 3.5 U of Expand High Fidelity enzyme mix (Roche S.p.A., Milan, Italy). To limit the possible PCR artifacts for each analyzed alpaca, three-four white colonies were selected from at least three independent RT-PCR reactions and sequenced on both strands.

### 2.4. Amplification of the* MC1R* Coding Sequence from DNA

The amplification of the complete coding sequence was performed using the MCRF3 and MCR5R1 primers (Table 1) in a 50 *μ*L reaction volume containing 1 × Expand High Fidelity PCR buffer (1.25 mM MgCl_2_), 0.3 mM dNTP, 0.3 *μ*mol of each primer, and 3.5 U Expand High Fidelity enzyme mix (Roche S.p.A., Milan, Italy) with the following cycling conditions: initial denaturation at 95°C for 3 min, followed by 35 cycles of 95°C for 30 s, 64°C for 30 s, and 72°C for 1 min, with a final extension at 72°C for 7 min. Three white colonies were selected from at least three independent PCR reactions and sequenced on both strands.

### 2.5. Cloning and Sequencing of the PCR Products

The PCR products were electrophoresed on a 1.2% agarose gel. The amplified fragments were gel-eluted using a NucleoSpin gel extraction kit (Qiagen, Milan, Italy) according to the manufacturer's instructions. The purified products were then ligated into the PGEM-T easy vector system (Promega, USA) according to the manufacturer's instruction. Approximately 5 *μ*L of the ligated products was transformed into DH_5_ *α E. coli* competent cells. Transformed colonies were selected using the blue-white colony screening method and sent to BMR Genomics, Italy, and StarSeq, Germany, for sequencing.

### 2.6. Sequence Analysis and Alignment

Nucleic acid and protein database searches were performed using BLAST from the NCBI server. The cDNA sequence data were analyzed using DNASTAR 5.0 [24]. Alignment of MC1R protein amino acid sequences proteins was performed using ClustalW [25]. The mRNA motif and secondary structure predictions were performed using RegRNA [26]. In silico functional analysis of missense mutations was obtained using PANTHER [27] and SNP tool [28].

### 2.7. Expression of Alpaca* MC1R* in Skin and Statistical Analysis

To detect differences between* MC1R* mRNA expressions in white, black, and brown alpacas, we performed RT-PCR analysis using a pair of* MC1R* gene-specific primers (MC1RFw and MCR5R2). Equal amounts (2 *μ*g) of total RNA extracted from skin biopsies of white (*n* = 5), black (*n* = 5), and brown (*n* = 5) alpacas were reverse-transcribed into cDNA using Takara reverse transcriptase following the manufacturer's instructions (Takara Biotech, Japan). Synthesized cDNAs were used as templates for RT-PCR reactions with the following conditions: initial denaturation at 95°C for 3 min, followed by 30 cycles at 95°C for 40 s, 60°C for 30 s, and 72°C for 15 s followed by a 7 min incubation at 72°C. A pair of primers (GAPFw and GAPRv) (Table 1) was used to amplify constitutively expressed glyceraldehyde 3-phosphate dehydrogenase (*GADPH*) gene cDNA as an internal control using the PCR conditions mentioned above. Identical volumes of the PCR products were applied to a 1.5% agarose gel, stained with ethidium bromide, and evaluated by band densitometry using Qscan 3.0 software. All reactions were carried out in three independent experiments. The relative levels of gene expression were analyzed* via* one-way ANOVA (analysis of variance) and are shown as the mean ± SD. Individual mean comparisons were performed using Duncan's test. Differences of *P* < 0.05 were considered significant. All statistical analysis was carried out using BioEstat v.5.3 [29].

## 3. Results and Discussion

### 3.1. Cloning and Characterization of* MC1R* Transcripts

**Figure 1:**
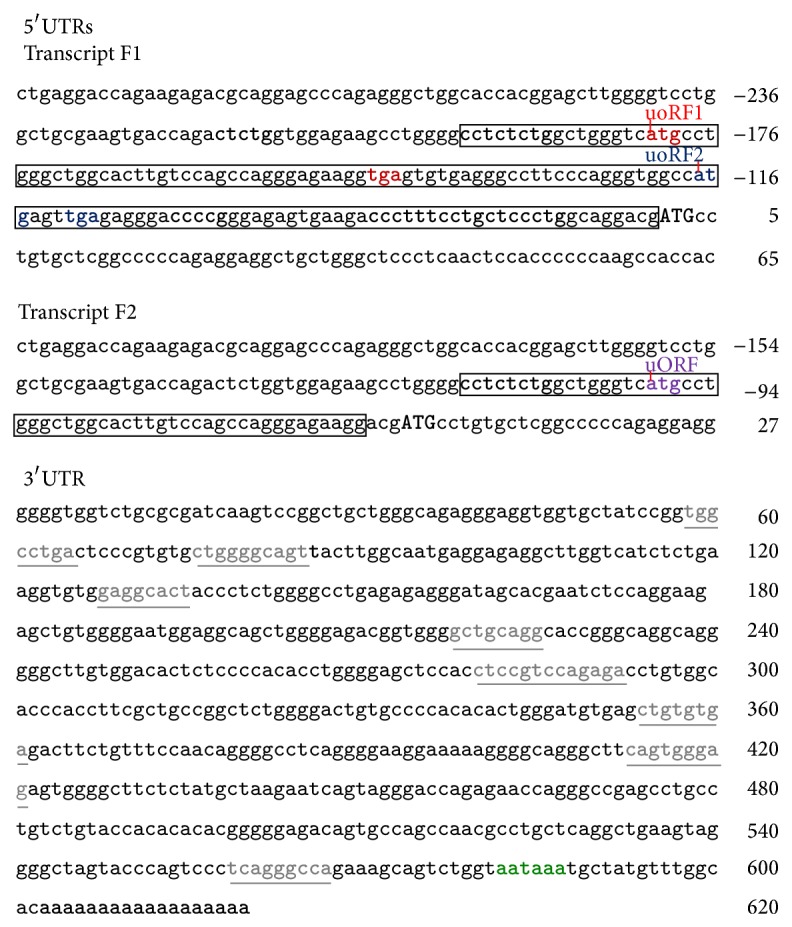
5′UTR and 3′UTR nucleotide sequences of* MC1R* transcripts. The two different 5′UTR nucleotide sequences (transcripts F1 and F2) and the common 3′UTR of MC1R transcripts are presented separately under the appropriate headings. The predicted TOP regulatory motifs and IRES in 5′UTR are indicated in black bold letters and box, respectively. The uORF start codons (atg) in the 5′UTR sequences are underlined and named immediately and the various uORFs and STOP codons sharing the same frames are colored identically. In all transcripts the main ORF is indicated in uppercase and bold letters. The miRNA seed sites in the 3′UTR are underlined and colored identically. The poly-A signaling sequence (aataaa) is in bold and green colored and the poly-A tail sequence is in bold in the 3′UTR sequence.

We performed RACE experiments using total RNA isolated from white and pigmented (brown and black) alpaca. Sequence analysis revealed two types of transcripts hereafter named F1 and F2 of 1810 and 1728 bp, respectively. The F1 and F2 transcripts possess an open reading frame (ORF) of 954 bp, a common 3′UTR of 602 bp, and they differed exclusively in the length of their 5′UTRs of 236 and 154 bp, respectively (Figure 1) (GenBank accession numbers HQ645018 and HQ645019). The 5′UTR of the shorter F2 transcript had an 82 bp deletion at the 152–233 bp position (Figure 1). Blast analysis of the F1 5′UTR against the 2X genome of alpaca in Ensembl showed that this sequence was identical to the corresponding genomic DNA. The main characteristics of F1 are the presence of a predicted internal ribosome binding site (IRES), which mediates translation initiation using an internal ribosome binding mechanism [30, 31], of five TOP regulatory motifs which play a critical role in the translational coordination control mechanism [32], and of three AUGs. ORFs have been shown to function as cis-acting regulatory signals that are able to moderate expression of the downstream reading frame [33]. The shorter F2 transcript includes a uAUG, an IRES of 28 bp, and three TOP regulatory motifs. The features observed in the F2 5′UTR could portray a nonfunctional mRNA. It has been reported that translation is severely hampered in long 5′UTRs containing AUGs, uORFs, and/or secondary structures [34]. Alternative mRNAs differing only in their 5′UTR are quite common and their expression may be regulated through alternative promoter usage [35, 36]. Interestingly and similarly to alpaca* agouti* transcripts [21],* MC1R* transcripts appear to have color specific expression as F2 transcripts have only been identified in white and not colored alpaca.

The common 3′UTR had a typical polyadenylation signal (AATAAA) followed by an additional 18 bp poly-A tail and eight microRNA seeds (Figure 1) as predicted by RegRNA. The fact that many microRNAs have short, perfect seeds of at least 6–8 bases near the 5′ end of the microRNA that are complementary to sequences within the 3′UTRs that can regulate translation [37–39] is established. 3′UTR elements may also control mRNA subcellular localization, stability, and translation efficiency [40, 41]. Further studies are required to investigate the predicted motif and to validate the regulatory functions of the observed 5′UTRs and 3′UTRs.

### 3.2. Polymorphisms in MC1R

To analyze* MC1R's* association with coat color in Peruvian alpaca, a panel from the segregation analysis of DNA from three different solid colored alpaca (black 17, brown 15, and white 15) was screened for polymorphisms. In our analysis, there were a total of twelve SNPs; among those four were silent mutations (c.126C>A, c.354T>C, c.618G>A, and c.933G>A), five were missense mutations (c.82A>G, c.92C>T, c.259A>G, c.376A>G, and c.901C>T) (Table 2), and three were from the 3′UTR region (c. ^*^5T>C, c. ^*^166C>T, and c. ^*^398G>A) and there was a four-base pair deletion (c.224_227del) (Table 2). Since the mutations resulting in an amino acid sequence change could possibly be causative for coat color variation, a further analysis of the missense mutations was conducted by means of the SNP annotation tools (cSNP and SNAP tool) to evaluate if the identified mutations may produce deleterious effects on the stability and function of the protein (Table 2). Hence the amino acid changing mutations were further considered for the association analysis. The missense mutations observed in the study were genotyped by direct sequencing in our segregation analysis samples and individual genotypes with phenotypes were compared for coat color association. The c.901C>T nucleotide mutation resulting in the p.R301C amino acid change (Table 2) showed significant correlation with the brown phenotypes of our population [18]. A similar type of mutation (C901T or chestnut) was also observed in horse [42]. In our population, 15 out of 17 black animals were homozygous for the C901 mutation and two were heterozygous for C901T. All the brown animals analyzed in the present study were heterozygous for C901T. And 14 out of 15 white animals were homozygous for T901 and one was observed to be heterozygous for C901T. The C-terminus of a GPCR is a functionally important domain involved in ligand receptor complex interactions with G-proteins, placing the receptor within the membrane and providing signals for its intercellular trafficking [43, 44]. Moreover, c.901C>T is near a potential phosphorylation site and mutation of this domain is reported to impair function [43, 44]. Interestingly in our molecular segregation analysis, animals homozygous for the mutation combinations A82/A259/A376/C901 (Table 3) expressed black phenotypes. The combination of G82/G259/G376/T901 mutations was observed (Table 3) to have white phenotypes. Brown phenotypes were observed to have a heterozygous condition for the following observed mutations: A82G/A259G/A376G/C901T.* In vitro* and* in vivo* functional analyses are needed to further confirm the effect of combinatorial mutations on phenotypes. In some species there are several alleles at the MC1R locus, with varying effects on phenotypes. A functional* MC1R* allele can lead to eumelanin production depending upon which allele is present at the agouti locus. Nonfunctional* MC1R* alleles result in nonblack phenotypes by preventing MSH from binding to* MC1R*. This loss of function can cause a range of phenotypes from red to as light as white as reported in the black bears [45]. Some species have a dominant black allele that allows MSH to bind to MC1R even in the presence of agouti. In pigs, there are 7* MC1R* alleles with 4 distinct phenotypes [42] and in humans 30* MC1R* alleles with only 2 phenotypes have been reported [46]. A similar association has been found between* MC1R* nonfunctional homozygotes and a red phenotype in many species including horses, dogs, and cattle [7, 12, 47]. Hence, the screening of these mutation combinations may better unveil the* MC1R* background for the selective breeding of alpaca.

### 3.3. Structure of MC1R

**Figure 2:**
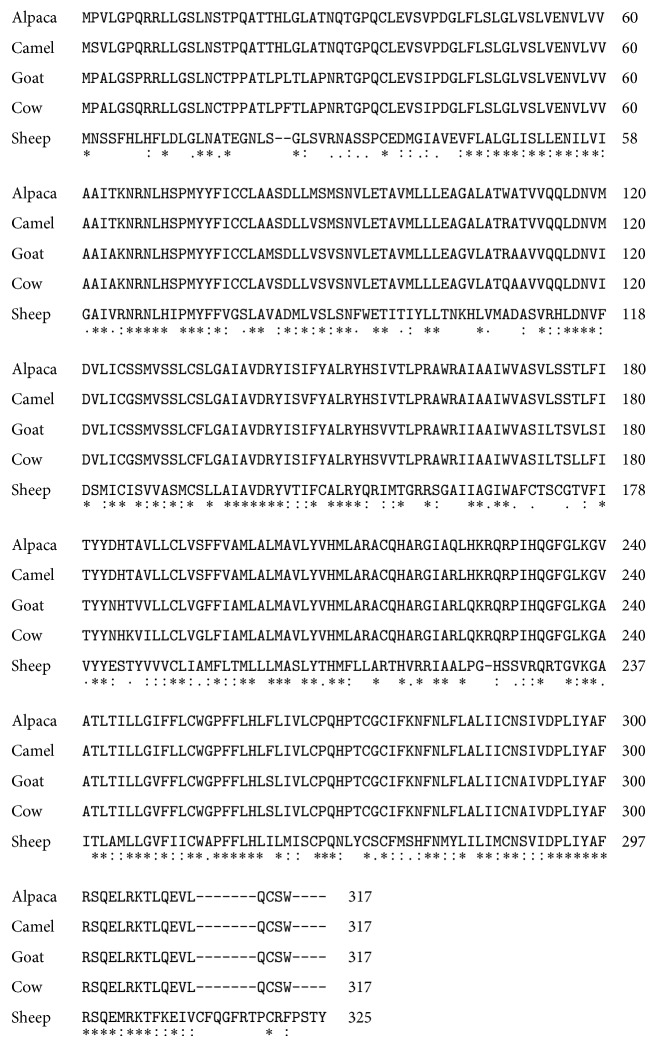
ClustalW alignment of MC1R amino acid sequences from alpaca, camel, goat, cow, and sheep shows the degree of identity.

The amino acid sequence deduced from the* MC1R* cDNA sequence showed an ORF of 954 bp and was found to encode a putative protein containing 317 amino acid (aa) residues with an estimated molecular mass of 35006.95 daltons. The amino acid sequence of alpaca MC1R was compared with other known MC1Rs; the results indicated that the amino acid sequence of alpaca MC1R shared high identity with that of camel, sheep, goat, and cow 97%, 89%, and 88%, respectively (Figure 2).

**Figure 3:**
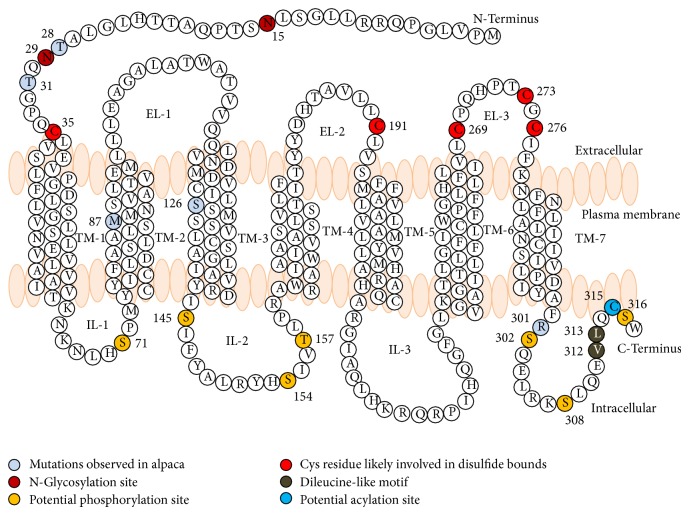
Structure of the alpaca MC1R. N-Terminus, extracellular loops (Els), intracellular loops (ILs), transmembrane (TM) regions, and the cytosolic C-terminal extension are labelled. The potential posttranslational modification sites and mutations reported in alpaca are also highlighted.

The hypothetical structure of alpaca MC1R was highly conserved among mammals including the N-terminus, extracellular loops, intracellular loops, transmembrane regions, and the cytosolic C-terminal extension. Comparative analysis of human and alpaca MC1R revealed the position of an N-glycosylation site, a potential phosphorylation target, Cys residues for disulfide bonds, a dileucine-like motif, and a potential acylation site [43] that were highly conserved. In alpaca, 10 mutations in the CDS have been reported for the* MC1R* gene ([18, 19] and our study); among those mutations 6 (Table 2) have been reported as amino acid changing mutations. This polymorphic condition within the population shows that alpaca may be under selective pressure and the polymorphisms reported in the locus do not affect the potential posttranslational modification sites (Figure 3). The occurrence of synonymous and nonsynonymous polymorphisms without functional implications at various regions of the gene indicates the maintenance of structural integrity and regulation despite selection pressure. Functional analysis of MC1R with mutations in the potential posttranslational modification site may give more insight into the function behind this.

### 3.4. *MC1R* Expression in White, Black, and Brown Alpacas

**Figure 4:**
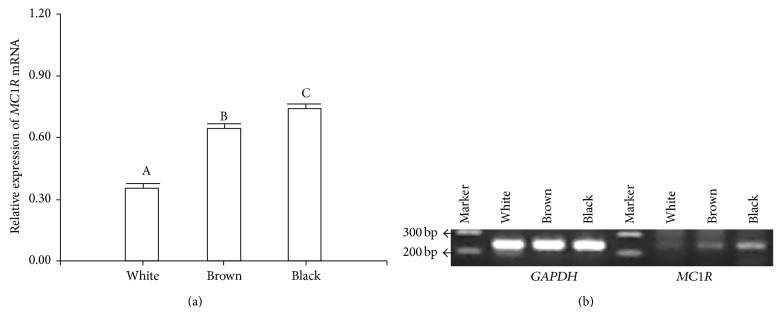
Gene expression of* MC1R* mRNA in white, brown, and black alpaca. (a) Relative expression of* MC1R* transcripts was measured using RT-PCR methodology and normalized against the reference gene* GAPDH*. Data are shown as the mean ± SE (*n* = 5) (*P* < 0.05). The distinct capital letters above the bars indicate a statistical significance among white and color morphs. (b) An ethidium bromide stained gel of* MC1R* and* GAPDH* amplicons.

To identify possible difference(s) between expression levels* of MC1R* mRNA in white, black, and brown alpacas, a total of 15 randomly chosen animals (5 for each phenotype under study) were analyzed using RT-PCR approaches. A 240 bp* MC1R* fragment and a 250 bp* GAPDH *gene fragment were amplified from each of the total RNAs extracted from the white and colored phenotypes; subsequently their expression levels were compared between the phenotypes. Analysis of variance showed that* MC1R* expression significantly varied with color (Figure 4(a)). The expression levels were comparatively high in black (0.76 ± 0.03), moderate in brown (0.65 ± 0.04), and low in white (0.38 ± 0.04) skin biopsies (Figure 4(b)). This assay shows that* MC1R* transcript levels are upregulated in the skin of black alpacas. Increased MC1R expression enhances the production of eumelanin, while diminished MC1R activity results in the production of pheomelanin [5, 6]. MC1R is located specifically on melanocyte membranes [2, 48–50] where it functions by switching the type of melanin produced from the red/yellow pheomelanin to the black/brown eumelanin [43, 51]. Our results suggest that eumelanin synthesis is dependent on MC1R expression levels [34] and may therefore enhance cell sensitivity to melanogenic stimuli.

In conclusion, the genetic dissection of* MC1R* in alpaca is the first step for development of marker based selection for coat color. The alleles identified in pheomelanic and eumelanic individuals could be used as markers for animal selection in breeding programs. Moreover, the results presented here refine the existing knowledge on the melanogenesis pathway and could also help in understanding its regulatory mechanisms.

## Figures and Tables

**Table 1:** Primer sequences used in coding sequence amplification, 5′ and 3′ RACE experiments, and expression analysis of *MC1R* gene.

Primer name	Primer sequence (5′-3′)
MCRF3	A TGCCTGTGCTCGGCCCCCAGAGGA
MCfw	GGCTCCCTCAACTCCACC
MC1RFw	AGACCCTTTCCTGCTCCCTG
MCR5R1	TCACCAGGAGCACTGCAGCACTTC
MCR5R2	GTTCTCCACGAGGCTCACCAG
MCR5R3	GCAGCAGATGAAGTAATACATGGGAG
GAPFw	ATCACTGCCACCCAGAAGAC
GAPRv	CTGCTTCACCACCTTCTTGA
O dTmodi	GAGAGAGAGAGAGACAGAGAACTAGTCTCGAGTTTTTTTTTTTTTTTTTT
NSTodt	GAGAGAGAGAGAGACAGAGAACTAGTCTCGAG
SA	AAGCAGTGGTATCAACGCAGAGTGNNNNN
ASA	p-ACTCTGCGTTGATACCACTGCTT (5′-phosphorylated)

**Table 2:** Mutations observed in *MC1R* of Peruvian alpaca.

SNP observed	Amino acid change	Effect on protein due to amino acid change
c.82A>G	p.T28A	Polar to nonpolar
c.92 C>T	p.T31M	Polar to nonpolar
c.126C>T	No change	NA
c.224_227del	Frame shift	Frame shift
c.259C>T	p.M87V	Nonpolar to polar
c.354T>C	No change	NA
c.376A>G	p.S126G	Polar to nonpolar
c.618G>A	No change	NA
c.901G>A	p.R301C	Polar to slightly polar
c.933G>A	No change	NA
c.^*^5T>C	NA	NA
c.^*^166C>T	NA	NA
c.^*^398G>A	NA	NA

NA: not applicable.

**Table 3:** The *MC1R* genotypes and phenotype of Peruvian alpaca.

c.A82G (p.T28A)	c.A259G (p.V87M)	c.A376G (p.G126S)	c.C901T (p.R301C)	Color	Number of animals	Proposed *MC1R* alleles
A/A	A/A	A/A	C/C	Black	15	*EE *
G/G	G/G	G/G	T/T	White	14	*ee *
A/G	A/G	A/G	C/T	Brown	15	*Ee *
A/G	A/G	A/G	C/T	Black	01	*Ee *
A/G	A/G	A/G	C/C	Black	01	*Ee *
G/G	G/G	G/G	T/C	White	01	*Ee *
